# Engineered Biofilm: Innovative Nextgen Strategy for Quality Enhancement of Fermented Foods

**DOI:** 10.3389/fnut.2022.808630

**Published:** 2022-04-11

**Authors:** Sreejita Ghosh, Moupriya Nag, Dibyajit Lahiri, Tanmay Sarkar, Siddhartha Pati, Zulhisyam Abdul Kari, Nilesh P. Nirmal, Hisham Atan Edinur, Rina Rani Ray

**Affiliations:** ^1^Department of Biotechnology, Maulana Abul Kalam Azad University of Technology, Haringhata, India; ^2^Department of Biotechnology, University of Engineering & Management, Kolkata, India; ^3^Department of Food Processing Technology, Malda Polytechnic, West Bengal State Council of Technical Education, Government of West Bengal, Malda, India; ^4^NatNov Bioscience Private Limited, Balasore, India; ^5^Skills Innovation & Academic Network (SIAN) Institute, Association for Biodiversity Conservation and Research (ABC), Balasore, India; ^6^Faculty of Agro Based Industry, Universiti Malaysia Kelantan, Kota Bharu, Malaysia; ^7^Institute of Nutrition, Mahidol University, Salaya, Thailand; ^8^School of Health Sciences, Health Campus, Universiti Sains Malaysia, Kubang Kerian, Malaysia

**Keywords:** food science, exopolysaccharide, fermentation, food biotechnology, future food

## Abstract

Microbial communities within fermented food (beers, wines, distillates, meats, fishes, cheeses, breads) products remain within biofilm and are embedded in a complex extracellular polymeric matrix that provides favorable growth conditions to the indwelling species. Biofilm acts as the best ecological niche for the residing microbes by providing food ingredients that interact with the fermenting microorganisms' metabolites to boost their growth. This leads to the alterations in the biochemical and nutritional quality of the fermented food ingredients compared to the initial ingredients in terms of antioxidants, peptides, organoleptic and probiotic properties, and antimicrobial activity. Microbes within the biofilm have altered genetic expression that may lead to novel biochemical pathways influencing their chemical and organoleptic properties related to consumer acceptability. Although microbial biofilms have always been linked to pathogenicity owing to its enhanced antimicrobial resistance, biofilm could be favorable for the production of amino acids like l-proline and L-threonine by engineered bacteria. The unique characteristics of many traditional fermented foods are attributed by the biofilm formed by lactic acid bacteria and yeast and often, multispecies biofilm can be successfully used for repeated-batch fermentation. The present review will shed light on current research related to the role of biofilm in the fermentation process with special reference to the recent applications of NGS/WGS/omics for the improved biofilm forming ability of the genetically engineered and biotechnologically modified microorganisms to bring about the amelioration of the quality of fermented food.

## Introduction

Generally, bacteria remain attached to surfaces to form a structured organized network within a self-generated matrix made of exopolysaccharide (EPS) to form biofilms (1–4). Over a long period of time, this sessile bacterial life form has served as a well-organized survival strategy for microbes due to the protective shield formation and physiological modifications brought about by the matrix of the biofilm while fighting against external stress conditions typically faced by bacteria in both natural and man-made settings. In case of pathogenic bacteria, biofilm formation is one of the most significant reasons for medical infection development, which is not easily cleared (5). Type I fimbriae is one of the important components leading to biofilm formation in Gram negative bacteria *Escherichia coli* (6). However, these structures or factors responsible for biofilm formation can be biologically engineered and used as a component to enhance fermented food quality. Biofilm possesses various beneficial activities in the field of agriculture and the food industry (7). The presence of high biomass within the biofilm plays a vital role in various types of biochemical activities (8). The industries at present are involving various engineered biofilm for the development of various types of products (9). There are various favorable properties of biofilm that can be used effectively for various applications. In various form, the biofilms are considered in fermented food formation, e.g., cheese, vinegar, kombucha, kefir, wine, lambic beer, miso, and kimchi (10).

On the other hand, fermentation leads to the disintegration of organic macromolecules through microbial activity into simple molecules. For example, enzymes from yeast convert starch and sugar to alcohol whereas the proteins get converted to amino acids/peptides. The microbial enzymatic activities on food components lead to food fermentation producing desirable biochemical alterations which lead to the noticeable modification of the food. Fermentation is a natural process of improving essential amino acids, vitamins, proteins, anti-nutrients, flavors, food appearance, and enriched aroma. Thus, fermentation is a process to reduce the energy required to cook and make the food safer. Hence, microbial actions play an important role in food fermentation by enhancing both the physical as well as the chemical properties of food. Fermented foods have longer shelf life, improved organoleptic properties, eliminated unwanted/ harmful components from food and improved antioxidant properties. Examples of fermented foods include cheese, garri, bread, ogi, milk, and yogurt.

Substrate composition and fermented microbes are the two main factors influencing fermented food quality. The treatment of food and the period of fermentation during food processing also influence fermented food quality. For most of the fermented beverages and foods, lactic acid bacteria (LAB) have been found to be the most dominant group of microbes and they impart beneficial effects to the fermented foods (11). The group of LAB mainly consists of *Streptococcus, Enterococcus, Lactobacillus, Leuconostoc*, and *Pediococcus* and also yeasts and molds such as *Kluyveromyces, Debaryomyces, Geotrichum, Saccharomyces, Penicillium, Mucor*, and *Rhizopus* species (12).

Over the last decade, next generation sequencing (NGS) has evolved from being just a tool for research to becoming widely used in diverse fields such as the investigations of outbreaks, diagnostics, forensics, antimicrobial resistivity, and authenticity of food. This tool is developing very rapidly with increased developments in food quality as well as making the food cost-effective, and it has a great influence on food microbiology. Food microbiology uses NGS in 2 different ways which are whole genome sequencing (WGS) where full length genome sequence determination of a single isolated culture (for example, a virus or a colony of bacteria or any other organism) is done and metagenomics, in which NGS is being applied to any biological sample for multiple sequence generation of all/most of the microbes present within that sample. The high discrimination capability of WGS in comparison with conventional molecular typing methods is well-known and thus, WGS is increasingly recognized as an innovative surveillance research tool for food borne diseases (13). WGS method is rapidly replacing conventional microbial characterization and typing tools thereby providing a more precise and faster result. The application of metagenomics for the improvement of food quality and safety is still in its budding stage and provides innovative opportunities for engineering microbial biofilms and predicting changes in microbial communities and can also help in unknown microbiota characterization.

## Microbe-Assisted Nutritional Quality Improvement in Fermented Foods

Fermented foods are known to have more nutritional values than their unfermented equivalent (14). The added nutritional value of fermented foods is because of the presence of fermenting microbes. The different fermentation processes are either anabolic (breaking down of complex or large compounds) or catabolic (synthesis of complex vitamins and various growth factors) in nature.

Substances that are not digested free the nutrients, which remain locked inside plant cells and structures. This mainly takes place in case of individual grains and seeds. During the process of milling, hemicellulosic, and cellulosic structures that surround the endosperm (enriched with digestible carbohydrates and proteins) are mechanically ruptured for releasing the nutrients. Crude milling is employed in lesser developed regions for nutritional content extraction but it is not sufficient to release all the nutrients from the products of the plants. Subsequent to cooking, some of the blocked nutrients still stay inaccessible to the digestive system in humans. Thus, this problem can be solved by the use of some yeasts, molds, and bacteria, which can break or decompose indigestible coverings and the cell walls of such products chemically as well as physically (14).

Enzymatic polymer degradation is another way of increasing the nutritional properties of plant materials. In this process, the polymers, which cannot be digested by humans into simple sugar derivatives such as hemicelluloses, cellulose, and other polymeric forms, are degraded by means of enzymatic actions (15). By means of the enzymes produced by the microorganisms, substrates rich in cellulose within fermented foods may be improvised for the consumption of humans. There exist cereals, which are low in nutritional components and are also consumed as an important staple food by the poor. Yeast and LAB fermentation were found to increase the nutritional value and digestibility of food. Yeast biofilm plays an important role in the fermentation of the table olives with the development of various sensorial features. The biofilm formed by yeast plays a pragmatic role in the process of debittering of the olives in the presence of the enzyme β-glucosidase. This enzyme further helps in the degradation of the phytic compounds with the release of various types of inorganic phosphorous (16). *Candida boidinii* shows co-aggregation with yeast on the olive surface along with the lactic acid bacteria (LAB) for the purpose of establishing polymicrobial biofilms. This further helps in the better fermentation of the olive oil (17–19). The yeast biofilm also plays important role in the dairy industry. The presence of various microbial communities plays an important role in influencing the quality of the dairy products both in positive as well as negative manner. It has been observed that *Kluyveromyces marxianus* isolated from the milk of goat has the potential to develop biofilm whereas the yeast helps in the maturation of it (20). Various types of alcoholic beverages also contain yeast biofilm which help in the mechanism of oxidative metabolism (21). The mechanism of ethanol oxidation to produce acetaldehyde in the presence of yeast acts as important precursor molecule in the development of some sensorial properties of aged wines (22).

The process of fermentation even increases microbial enzymatic activities by providing acidic conditions at a temperature around 22–25°C (23). A major function of enzyme-mediated hydrolysis within fermented food products involves the reduction of the content of anti-nutrients phytic acid (phytase-mediated degradation) and tannins increasing the bioavailability of polysaccharides (amylases) or simple sugars, free fatty acids (lipase), proteins (protease), and iron.

## Role of Biofilm in Media Lactic Acid Fermentation Upon Nutritional Qualities of Food

The biofilm lifestyle has significantly enhanced the quality of food since recently, it has gained a considerable amount of importance in the nutraceutical and pharmaceutical applications. The bioactive compounds are those present within the EPS that possess antimicrobial and antioxidant potentials along with the decrease of the level of cholesterol and act as prebiotic agents (24).

Hence, the production of exopolysaccharides by the group of LAB has attracted the food industry. The use of EPS as a thickening agent and bringing about rheological changes within the food has made it an important field of research (25). The composition of EPS varies greatly and has varied effect on human health. Potential groups of probiotic organisms have been observed extensively within various vegetables. The EPS produced by the sessile colonies help in providing various functional properties that includes the prevention of the adhesion of various groups of pathogenic organisms on animal cell lines (26). The EPS formed by the LAB act as alternative food additives due to the various types of physicochemical features exhibited by them. The rheological changes provided to the food by the EPS makes it an important field of research and utility for the food industries (27).

The biofilm embedded bacterial cells of *Lactobacillus plantarum* showed the ability of malo lactic fermentation and such efficacy for L-malic acid conversion is higher in the case of sessile cells than that of planktonic form, which favors the use of biofilm bound cells in wine manufacturing industries (28). Similarly, the biofilm bound cells of various LABs including *Lactobacillus casei* ATCC 334 showed improved survival ability under adverse conditions like low pH environment during malolactic fermentation (29).

**Figure 1:**
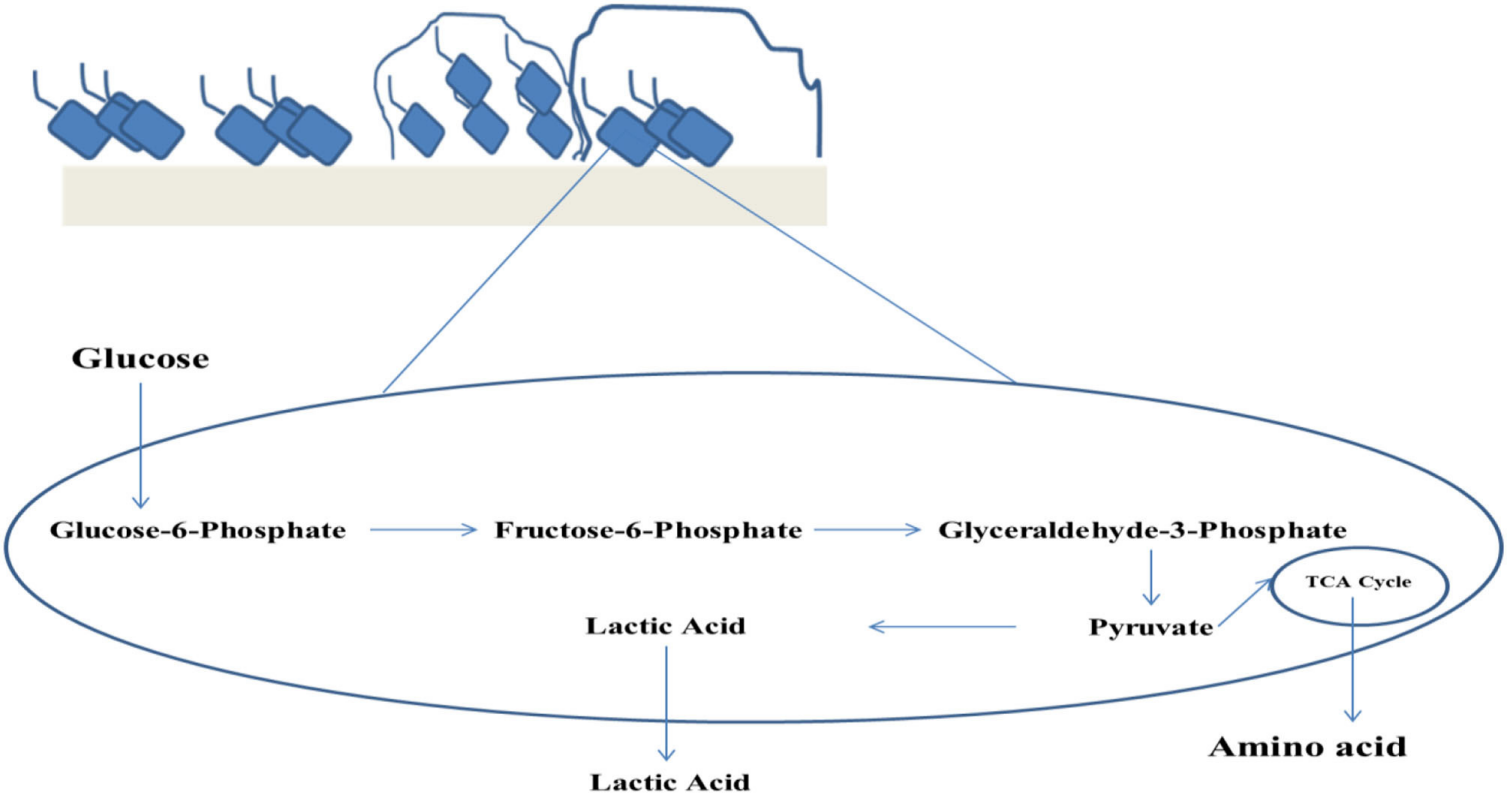
Biofilm mediated lactic acid fermentation.

The major parameters that impart the nutritional quality of food comprises of digestibility and the quantity of essential nutrients present in it. Both digestibility as well as nutritional aspects can be enhanced by fermentation. In the process of fermentation, fermenting microbial enzymes, at the beginning, start to digest the macronutrients (30). The various ways through which the nutritional quality improvement of food may be carried out by fermentation involve the increment of the quantity and the bioavailability of nutrients thereby escalating nutritional density. Nutritional density can be increased by promoter synthesis for the anti-nutritional component degradation absorption, thereby influencing the nutrient uptake by mucosa and the pre-digestive stages of the food constituents (31). The bioavailability of limiting amino acids and few micronutrients along with protein solubility can be improved by the process of lactic acid fermentation (32). Through this process, 90% of oligosaccharides, phytates, and 50% of tannins also get reduced (33) (Figure 1). This exerts an indirect or direct nutritional effect of fermented foods on nutrition-deficiency diseases. The direct effect of fermentation on food is the curative effect. Similarly, the fermentation of foods directly influences the health of the consumers by escalating the quantity of available vitamins like thiamine, niacin, riboflavin, or folic acid (34). This even increases the utilization of iron *via* breaking down complex materials into inorganic ions containing vitamin C (31).

The fermentation process in foods increases the bioavailability of trace elements as well as minerals by the reduction of indigestible material of plants like polygalacturonic and glucuronic acids, hemicelluloses and cellulose (35). It even diminishes serum cholesterol by the inhibition of cholesterol biosynthesis within liver and endogenous and dietary cholesterol absorption inside the intestine (36). It is stable, robust, and safe for the food product, thereby preventing infections or diseases such as salmonellosis and diarrhea (37).

## Alterations and Improvement of Biological Constituents of Fermented Foods

### Bio Enrichment With Vitamins

Studies have revealed that the biofilm formed by Marine Roseobacter Clade help in the production of essential vitamins and auxins (38). The biofilm present within the intestine also helps in the synthesis of vitamins like folic acid, biotin, and vitamin K (39). The biofilm constituted by the Lactobacillus app also help in the synthesis of vitamin K along with various types of water soluble vitamins comprising of pyridoxine, riboflavin, thiamine, cobalamin, folates, and thiamine (40).

A dish of Indonesian origin known as “tempe” can be prepared through soaking followed by dehulling and partial cooking of soya beans in the presence of some molds or *Rhizopus oligosporus* (41). This mold gets transformed to a solid cake by knitting the cotyledons in slices followed by cooking. During the process of fermentation, proteins get partially hydrolysed, lipids are hydrolysed to form stachyose (tetrasaccharide, which humans are unable to digest), riboflavin becomes increases two times, fatty acids and niacin increases 7 times along with vitamin B12, which is usually not found in vegetarian foods and is produced by fermenting bacterial growth in association with the necessary mold. The process by which tempe is manufactured decreases the time for cooking and enhances the digestibility as well as the texture of a large quantity of legume or cereal mixtures. A non-pathogenic strain of *Klebsiella pneumoniae* produces vitamin B12 on the inoculation fermentation process of Indian idli.

The fermentation of juices from Agave (cactus plant) leads to the production of Mexican pulque and the most ancient alcoholic beverage in the continent of America (42). Pulque is generally consumed by children from the lower-middle class families of Mexico due to its enrichment with thiamine, niacin, riboflavin, pantothenic acid, biotin, p-aminobenzoic acid, and pyridoxine. For example, an alcohol-containing beverage called Kaffir beer has a gruel and thin consistency with a sour but pleasant taste. Kaffir is a traditional beverage prepared by South African people with an alcohol percentage of 1- 8%. Kaffir is made from unmalted and malted kafficorn (*Sorghum caffrorum*). Kafficorn can be substituted with maize or millet. This beverage has a greater content of riboflavin and twice the quantity of nicotinic acid or niacin while the thiamine level is kept constant during the process of fermentation among people eating maize.

Palm sap is a plum, sweet, and milky white suspension of yeasts and bacteria that is a transparent and colorless liquid having almost 10–12% fermentable sugar. It is mostly consumed around the tropics. This class of wine contains ~83 mg/L ascorbic acid (43). Fermented palm wine contains an increased quantity of thiamine (from 25 to 150 μg/L), riboflavin (from 35 to 50 μg/L), and pyridoxine (from 4 to 18 μg/L). Astonishingly, there also exists a high amount of vitamin B12 (190–280 μg/L) in palm wine. Thus, palm toddies are considered as one of the cheapest vitamin B sources playing a major role in economically drained nutrition among the tropics.

### Enrichment With Antioxidants

The antioxidant activity of fermented foods comprises of 2-azino-bis (3-ethylbenzo-thaizoline-6-sulfonic acid, ABTS), reducing power assay, estimation of total phenol content (TPC) and 1,1-diphenyl-2-picryl hydrazyl (DPPH) radical scavenging potential. Several soybean fermented foods in Asia possess antioxidant activities such as bekang and tungrymbai (soybean fermented foods in India), tempe (mold fermented soybean food of Indonesia), kinema (soybean fermented food of Nepal and India), chungkokjang and jang (fermented soybean food of Korea), natto (fermented soybean food from Japan), thuanao (fermented soybean food from Thailand) and douche (soybean fermented Chinese food). Kimchi and yogurt also possess antioxidant activities. A pool of selected lactic acid bacteria was used for the sourdough fermentation of various cereal flours with the aim of synthesizing antioxidant peptides (44). Researchers have observed the antioxidant properties of LAB during skim milk fermentation by DPPH assay and found the scavenging potential of free radicals ranging between 14.7 and 50.8% (v/v) following fermentation up to about 24–72 h, respectively (45). It has been found from this study that various LAB species and the time of fermentation significantly influenced antioxidant properties (*p* ≤ 0.05). Antioxidant activity improvement in fermented sausage was carried out by bacteria *Lacticaseibacillus paracasei* (SR10-1) and *Lactobacillus curvatus* (SR6), which in turn leads to the overall enhancement of the safety and taste of the sausage (46). The reducing potential and DPPH scavenging activity was better in case of *L. curvatus* (SR6), i.e., 47.31% ± 4.62% and 59.67% ± 6.68% while anti-lipid peroxidation and OH scavenging capacities were better in case of *L. paracasei* (SR10-1) *viz*. 63.89% ± 0.93% and 285.67% ± 2.00%, respectively.

### Enrichment With Peptides

Proteinaceous components of the biofilm matrix include secreted extracellular proteins, cell surface adhesins (47). Bioactive peptides (BAP) are produced from proteolytic microbes (mainly *Bacillus*) when food gets fermented. Peptides possess anti-hypertensive properties and play a major role as immunomodulatory and anti-thrombic agents. Because of the lack of large-scale facilities for production and exorbitant costs of enzymes for hydrolysis of proteins, BAP production is yet to cross the benchmark. So, fermentation with microorganisms is a more cost-effective and economical method for BAP production. The health attributes of dairy products exist in BAPs. Till now, four various classes of cell envelope proteinases (CEP) have been found which are PrtP (produced by *L. paracasei* and *Lacticaseibacillus casei*), PrtB (produced by *Lactobacillus delbrueckii* subsp. *bulgaricus*), PrtH (produced by *Lactobacillus helveticus*), and PrtR (produced from *Lactiplantibacillus plantarum*) (48). Commonly, many species *Lactobacillus* contain a single CEP while *L. helveticus* contain four various paralogs of PrtH (PrtH4, PrtH3, PrtH2, and PrtH1), however the distribution of these CEPs are dependent on the strain of the bacteria. Because of the presence of many paralogs of CEPs and diverse specificities, *L. helveticus* is the species with the highest proteolytic capacity of the genus *Lactobacillus* and is significantly responsible for the various types of BAP production.

Inhibition activity of angiotensin-converting enzyme (ATE) was found in some of the previously fermented milk products like kefir, koumiss, cheese, yogurt, fermented fish products, and fermented camel milk (49). BAPs are also generated by soybean product fermentation, which help in the treatment and prevention of different metabolic diseases.

### Microbial Enzyme Production

Enzymes like proteinase, amylase, catalase, mannase, and cellulase are usually produced by fermenting microbes mainly, *Bacillus* in fermented soybean products (complex substances get hydrolysed to simpler biomolecules) of Asia. Enzymes that produce carbohydrates include α-amylase, amyloglucosidase, pectinase, maltase, cellulase, invertase, lipase, alkaline proteases, and β-galactosidase, which are generated from the mycelia fungi like *Actinomucor, Amylomyces, Mucor, Aspegillus, Rhizopus, Monascus*, and *Neurspora* in fermented beverages and foods. Enzyme produced from *Aspergillus oryzae* in koji (Taka amylase A, TAA) has many industrial applications. In the regions around the Himalayas, dry, stable, and cake-like amylolytic starter culture is used in alcohol production (50). Such starter cultures contain mixed strains of yeasts such as *Saccharomycopsis fibuligera, Saccharomycopsis capsularis*, and *Pichia burtonii* thereby increasing the amylase content (Table 1).

**Table 1:** Microbial enzymes having utility at various food processing industry.

**Microbial source**	**Enzyme produced**	**Application**	**Industry**	**References**
Bacteria, Fungi, Yeast	Amylase, protease, pectinase, invertase	Helps in clarification, filtration, pressing	Wine industry	(125)
Fungi	Amylases	Helps in the purpose to liquefy	Vegetables	(126)
Fungi	Amylases	Making of bread	Milling and baking Industry	(125)
Bacteria and fungi	Protease and amylase	Chillproofing and mashing	Beer industry	(125)
Fungi	Glucose oxidase	Helps in the mechanism of removing oxygen	Carbonated beverages	(127)

Nattokinase enzyme generated by *Bacillus subtilis* found in natto was studied for its fibrinolytic capacity (51). Few other strains of bacteria found from fermented foods include *Vagococcus carniphilus, Bacillus amyloliquefaciens, Pediococcus acidilactici, Vagococcus lutrae, Enterococcus faecium, Enterococcus faecalis*, and *Enetrococcus gallinarum* also possess fibrinolytic activities (52). *Virgibacillus halodenitrificans* strain SK 1-3-7 found from fish sauce exhibited fibrinolytic properties (53).

### Increased Production of Poly-Glutamate and Isoflavone and Saponin Values

Isoflavones like glycitein, genistein, and daidzein are produced by 4 chemical components of soybean which are aglycones, β-glucoside, acetylglucoside, and malonylglucoside. Some of the fermented products of Asia like sufu and douche (China), miso and natto (Japan), thuanao (Thailand), chungkokjang and doenjang (Korea) and tempe (Indonesia) hydrolyze glucosidic isoflavones to their corresponding aglycones on fermentation. Isoflavone (mainly Factor-II) and aglycone content increased after tempe fermentation. Isoflavones present in doenjang also increased the activation of the LDL-C receptor, which can prevent vascular diseases.

Saponins present in soybean are naturally glycosidic oleanane triterpenoids, which can be further classified into 2 classes, i.e., Group A, Group B, and Group E saponins and 2, 3-dihydro-2, 5-dihydroxy-6-metyl-4*H*-pyran-4-one (DDMP). Some of the beneficial health effects of Group E, Group B, DDMP and their derivatives, include the suppression of peroxidation capacity of lipids, reduction in proliferation of carcinogenic cells and help in avoiding hypercholesterolemia. Saponin content in natto increases after fermentation with *B. subtilis* (54). It was also found that a greater content of Group B saponin was present in kinema, which indicates that it exhibits good qualities to customers.

Poly-glutamic acids (PGA) is produced from various strains of *Bacillus* sp. such as *B. subtilis* and *Bacillus licheniformis* in fermented soybean products of Asia and is not produced from the ribosomal proteins. Viscous components of the fermented products of soybean in Asia are water-soluble, biodegradable, and non-toxic for human consumption.

### Degradation of Anti-nutrients

Anti-nutrients are present in almost all of the food substances. They are toxic for human consumption and limit the bioavailability of the essential nutrients to the human body by reducing the nutritive value of food. Anti-nutrients are degraded by microbes within fermented food products thereby making these inedible products consumable. Other processes responsible during processing are dewatering, grating, peeling, washing, roasting, and fermentation and all these steps diminish the content of cyanide in the final products in different fermented products of cassava in Africa such as fufu and gari. In some types of cassava, tubers are bitter because of the existence of cyanogenic glycosides linamarinis and lotaustralin, which get detoxified during conventional gari and fufu production by *Streptococcus, Lactobacillus*, and *Leuconostoc* and produce hydrocyanic acid having a low boiling point, which boils out while toasting the dewatered pulp thereby making the finished product safe and non-toxic for human consumption (55). *R. oligosporus* in tempe eliminates flatulence from non-digestible oligosaccharides like stachyose and verbascose and transforms them to disaccharides and monosaccharides, which are easily absorbed. Anti-nutritive substances from kinema are removed by *B. subtilis* through the reduction of phytic acid during rabadi and idli fermentation.

### Biochemical Alterations in Fermentation of Cereals

Cereals are the most important sources of dietary proteins, carbohydrates, fibers, minerals, and vitamins among the world. However, the disadvantage of cereals exists in their way of acceptance by consumers on the basis of its nutritive quality and sensory attributes of these products in comparison to milk products and milk. This is because of their low protein content, absence of essential amino acids (such as lysine), low bioavailability of starch, presence of anti-nutritive compounds (like tannins, polyphenols, phytic acid) and the coarse texture of the cereals.

Numerous methods have been brought into practice for the elevation of the nutritional properties of the cereals. For example, enhancement *via* genetic alterations and amino acid supplementation with a greater concentration of amino acids or alternative rich protein sources like legumes or defatted oilseed meals of the grains of cereals. Additionally, various mechanisms of processing like cooking, sprouting, milling, and fermentation improve the nutritional properties of the cereals and among all these methods, fermentation is regarded as the best one. Natural cereal fermentation usually decreases carbohydrate content along with indigestible polysaccharides and oligosaccharides thereby enhancing the bioavailability of the vitamin B group and increases the biosynthesis of amino acids. Natural fermentation facilitates the enzymatic biodegradation of phytates by providing with optimum conditions of pH existing within a complex medium with multivalent cations like iron, zinc, calcium and magnesium. Reduction in phytate content increases the bioavailability of soluble iron, calcium, and zinc many times.

Post-fermentation, the impact on protein and amino acid levels becomes controversial like for example, concentration of existing tryptophan, methionine and lysine rises within cornmeal. Similarly, cereal fermentation like maize, millet, sorghum, and many other grains noticeably enriches protein quality along with an increase in lysine levels (56). In contrast to this, while observing the nutritive properties of sorghum kisra bread, no increased lysine content was found. There was increase in the content of tryptophan during the production of uji; however, a significant reduction was observed in the content of lysine (31). This indicates that the effect of fermentation on the nutritional properties of food remains unaltered but the proof for enrichment is quite visible. Thus, food product fermentation enriches and improves the texture, flavor, aroma, taste, and shelf-life of food.

Various volatile substances are generated in the fermentation of cereals thereby producing a mixture of flavors within the food product. Besides the flavors, aroma-generating substances such as diacetyl acetic acid and butyric acid helps in the upgradation of appeal of the fermented cereals.

The most significant component utilized in conventionally fermented beverages and foods prepared worldwide are cereals (rice, wheat, corn, or sorghum). Few of these are utilized as important dietary foods for human consumption while the others are used as colorants, spices, beverages and breakfast. In most of the fermented cereal food products, fermentation is carried out either by the use of natural or a mixed culture of yeasts, fungi, and bacteria. During the process of fermentation, these microbes act sequentially or in a parallel way thereby altering the dominant microbiota (31).

Common fermenting bacterial species include *Lactobacillus, Bacillus, Streptococcus, Micrococcus, Leuconostoc*, and *Pediococcus*. Commonly used fungal genera for fermentation are *Aspergillus, Cladosporium, Paecilomyces, Trichothecium, Penicillium*, and *Fusarium*. The most significant species of yeast is the *Saccharomyces* commonly used in alcohol production. The parameters that trigger the fermenting microbes to ferment beverages/foods are the activities of water, pH, temperature, salt concentration, and composition of the food matrix. Most commonly used microorganisms carry out fermentation by the use of LAB. This kind of lactic acid fermentation renders enriched nutritional properties, longer shelf—life, wide acceptance, and safety of the fermented foods (30). When cereals are fermented naturally, the cereals are first cleaned followed by soaking in water for some days, a series of microbial activities take place, where the predominant bacterial group present is LAB. In such type of fermentation, amylases manufacture sugar due to fermentation by LAB as an energy source. In addition to fermentation, some steps such as salting, size reduction, or heating also help in the final product formation (57).

During homofermentation, the only primary or end product after fermentation of glucose by few LABs like *Pediococcus, Streptococcus, Lactobacillus*, and some of the lactobacilli is lactate. In contrast to this, during heterofermentation, microbes like *Weisella, Leuconostoc* and some lactobacilli, the end products consist of carbon dioxide, lactic acid, and ethanol. The technology of lactic acid fermentation also has a confirmed role in some of the cereal fermentation. Antibiosis using LAB was identified by organic acids, antibiotics, and hydrogen peroxide formation (58).

Organic acid production by LAB provides stress conditions for spoilage-causing microbes existing in cereals by pH reduction to <4. The acidic activity on the cytoplasmic membrane of bacteria gives rise to an antimicrobial activity thereby hindering membrane potential maintenance and affect active transport. Apart from production of organic acids, LAB also releases hydrogen peroxide along with flavin nucleotides, which quickly react with oxygen and oxidize the reduced nictotine amide adenine dinucleotide (NADH). It can even accumulate the enzyme catalase (since actual catalase is absent in LAB for hydrogen peroxide degradation) inhibiting the activity of several microbes (59). In cereals containing high amount of tannin, lactic acid fermentation may also decrease the level of tannin in some of the cereal crops for enhancing absorption of iron. LAB-mediated fermentation provides antitumor properties as well as viricidal effects (60).

Many legume food products consumed or produced in Africa and Asia were fortified by cereals for the improvement of the overall protein quality in fermented foods as legumes have higher lysine content but lack amino acids containing sulfur (31). On the contrary, cereals are enriched with cysteine and methionine but lack lysine.

### Biogenic Amine Production by LAB Fermentation in Vegetables and Juices

Biogenic amines (BA) are low molecular weight amines having well-known biological efficiency, which are mostly found in food items and beverages. However, the over-consumption of BA gives rise to psychoactive disorders and/or numerous vasoactive effects.

The spoilage of food during spontaneous and controlled fermentation generally BA concentration, where parameters such as pH, temperature, sodium chloride, or oxygen content affect BA formation adversely (61).

In most countries of Europe, sauerkraut, which is shredded white cabbage known for its sensory attributes and nutritional properties and is preserved by lactic acid fermentation. Sauerkraut production is a 3-step process and each is step is known for its acting microbial community producing BA. Some of the microbes involved in sauerkraut production are *Lactobacillus* sp., *Leuconostoc mesenteroides*, and *Saccharomyces cerevisiae*.

Researchers have conducted a study, where 121 sauerkraut samples were taken, in which the mean BA concentration was found to be 174, 146, and 50 mg/kg for tyramine, putrescine, and cadaverine, respectively (62). The histamine content was found to be <2 mg/kg in 44% of the total number of samples while the histamine value was 10 mg/kg in 19% of the samples. Researchers have observed that pasteurized and bottled German sauerkraut juices contain a huge quantity of putrescine up to 694 mg/dm^3^ (63). In another experiment the quantities of spermidine, cadaverine, putrescine, and tyramine were measured in different sauerkrauts prepared in the laboratory after storage of 6 months (64). A similar investigation was also carried out for determining BA content in cabbage juices formed by lactic acid fermentation (65). In red beet and carrot produced from lactic acid fermentation, histamine, cadaverine, spermidine, putrescine, and tyramine concentrations were found to be within 1–15 mg/ kg (66).

Researchers have formulated a sauerkraut with a less BA content under specific conditions such as temperature for initial fermentation fixed at 15–20°C and pasteurization started rapidly as soon as pH and acidity reached 3.8–4.0 and 9–10 g/kg, respectively to preserve it from bacteria (62). It was also observed that the content increases with pH ranging between 3.6 and 3.8 thereby indicating that pasteurization gets stopped once pH becomes <4 because at this point, the activity of yeast (*S. cerevisiae*) begins and increases the histamine content to 200 mg/kg (67). In another study it was found that when sauerkrauts were inoculated with a mixed culture of *L. plantarum, L. casei, Pediococcus* sp., and *E. faecium* or a single culture of *L. plantarum*, produces a low concentration of cadaverine, putrescine, and tyramine (64).

Every factor responsible for BA level reduction in production of sauerkraut leads to the primary stoppage of spoilage with monogenic bacteria in shredded cabbage, production machine for shredding, transporters, and silos. Researchers also observed that inoculants lacking amine are resistant against contamination (64).

## Increasing Nutritional Properties of Fermented Dairy Foods

The increased demand for fermented dairy products such as yogurt, sour milk, kumiss, curd, acidophilus milk and different milk products is because of their enriched nutritive values in comparison to normal milk. Even if the mineral composition of the fermented milk products remains unchanged, the quantity and quality of fats, carbohydrates, vitamins, and proteins get modified (68). The sour milk quality is dependent on the fermenting microbes and the end products produced by the biochemical reactions that take place during the process of milk souring. The end products consist of lactic acid, alcohol, carbon dioxide, antibiotics, and vitamins (69).

### Biochemical Processes for Enriching Nutritive Properties of Milk

#### Proteolysis Activity of Biofilm

Milk proteolysis breaks down proteins into amino acids, peptides, and peptones thereby magnifying the quantities of essential amino acids such as leucine, methionine, isoleucine, phenylalanine, tryptophan, tyrosine, threonine, and valine, which give specific health benefits to mainly persons, who are physically weak. Endo and exo peptides are responsible for carrying out proteolysis (70). Protein content rises from 85.4 to 90% in case of fermented milk like yogurt, curd, and kefir and has higher rate of digestibility of proteins because of the milk precipitation by lactic acid to fine curd thereby increasing the nutritional content. The free amino acid quantity of phenylalanine, proline, isoleucine, lysine, arginine, and cysteine increases during the process of fermentation followed by preserving the fermented milk. Because of the process of milk proteolysis and the biochemical alterations taking place within milk, milk products become very diet friendly (71).

#### Hydrolysis

Hydrolysis of lactose is carried out by few bacteria, which are already present inside milk. Lactose hydrolysis produces galactose (16–20%), glucose (0.6–0.8%) and lactose (45–50%) in comparison to an overall 5% of lactose in milk. LAB brings about lactose hydrolysis *via* β-galactosidase production. Lactose hydrolysis is actually essential for the production of lactic acid and in order to lower the pH of the bowel, inhibits the putrefaction of microbes during their growth. Lactic acid is also required to absorb calcium and organoleptic properties.

#### Lipolysis

Homogenization reduces fat globule size so that they are easily digested (72). Lipolysis causes physiological changes because of the increased content of fatty acid due to LAB.

#### Vitamin Alterations

The content of vitamins present in fermented milk is dependent on the type of bacterial culture within it. Vitamins such as ascorbic acid, B2, and B1 get reduced to almost half since they are used up by the bacteria growing in milk whereas vitamins like nicotinamide, riboflavin, and thiamine rise by 2 times (73).

#### Antibacterial Action

The antibacterial activity of fermented milk is dependent on the antibiotic action of the bacteria growing inside the milk like for example, lactobacilli is present in yogurt and there are also few other substances having antibacterial activities such as lactic acid, hydrogen peroxide, antibiotics, and bacteriocins (74).

#### Mineral Alterations

There is higher and increased mineral bioavailability in fermented milk especially potassium, calcium, magnesium, zinc, phosphorous, and potassium iodide because of the actions of LAB during and post-fermentation leading to acidity (75).

## Biochemical Modifications in Fermented Food Products Made of Meat

*Streptococcus, Lactobacillus, Leuconostoc, Pediococcus, Enterococcus*, and *Lactococcus* are some obligate/facultative anaerobic bacteria belonging to LAB (acidogenic) and gram-positive bacteria used for metabolization of saccharides with various extent of effectiveness into lactic acid, alcohols, amino acids, lipids, and aliphatic substances (76).

These microbes carry out 3 functions together in fermented sausage thereby releasing nitric oxide by nitrite and nitrate reduction, which gives the cured color after reacting with myoglobin and decrease the pH by DL-lactic acid production from glucose *via* anaerobic glycolysis (76).

Incubation of sausages in the presence of optimum temperature with obligate anaerobic conditions favor rapid LAB growth thereby transforming simple sugars to lactic acid and decrease the pH. The post-mortem range of 4.5–7 μmol/g is not enough to decrease pH. Hence, simple sugars need to be added as substrates for LAB and adjust the pH to 4.6–5. Researchers have utilized 0.62 g glucose/kg meat for reducing pH by 0.1 (77).

The main mechanism for metabolism of lactic acid involves carbohydrate fermentation in combination with the necessary extent of phosphorylation.

The homofermentative lactic acid fermentation pathway occurs *via* Embden-Meyerhoff-Parnas (EMP) pathway producing lactic acid as the only end product and gives a tangy sharp taste. Heterofermentative LAB even release lactic acid via 6-phosphogluconate/phosphoenoketolase pathway liberating a small quantity of acetic acid (10%), which is responsible for the off-taste of hexoses (78).

The main parameters in lactic acid fermentation are relative humidity, temperature, and time. For example, temperature and higher activity of water (a_w_) helps in rapid LAB growth reducing the pH. The smoke contributes to several aroma and antimicrobial substances like carbonyls, organic acids (formic, acetic, isobutyric, butyric, and propionic acids) and phenols (antioxidants) thereby coagulating superficial proteins and inhibiting microbes (79).

Change of pH can be detected by the conversion of lactate and ammonia to lactic acid. This conversion is carried out by carbohydrate addition from fermentation of glycerol with the help of ammonia and bacteria produced from fermentation of amino acids. The process of fermentation also leads to the production of acetic acid. Even, the oxygen that is used up in the process of metabolism and the conditions determining the microbial type and metabolism rates, affect the quantity of carbohydrates and lactose produced by fermentation (80).

Endogenous enzyme called cathepsin D present in muscles can break down myofibrillar protein to polypeptides having low pH. Proteolysis of proteins leads to the production of non-volatile and volatile flavors in fermented sausage resulting in peptides and free amino acids. *L. casei* and *L. plantarum* are responsible for the breakdown of aroma and flavor-producing compounds such as sarcoplasmic and myofibrillar proteins. For developing the fermented sausage structure, myofibrillar proteins are more significant than the sarcoplasmic protein.

In fermented meat-based products, lipolysis is carried out by the enzymes of the microbes present in muscular tissues, i.e., both exo- and endo-enzymes and fatty acid oxidation leads to the production of aldehydes, alkanes, alcohols, and ketones (81).

Oxidative and hydrolytic modifications are generally related to unsaturated fats and they are autocatalytic and they are also responsible for the production of flavors (both bad and good flavors) and aromas. Thiobarbituric acid reactive substances (TARS) are slightly increased in non-vacuum situations while it remains constant in vacuum conditions. Enzymes involved in lipolysis are naturally found in meat and they lead to the release of fatty acids whereas in fully cooked semi-dry products, the action is minimum. The ripening of mold and smoking are used in oxygen reduction *via* direct utilization and reduces the penetration of light. The diameter of the sausage also affects the biochemical processes, which are involved producing sausages with greater diameter (anaerobic) because of minimum oxidation. Glucose concentration does not affect the highest activity of lipases but it is required for the production of lipase (82).

There exist almost 200 volatile compounds that contribute to the aroma produced in fermented meat. Such compounds are produced from spices, smoking, acetoin, diacetyl (0.1 ppm), carbohydrate, and acetaldehyde (0.7- 1.5 ppm) metabolism. For example, aroma of vinegar results from acetic acid, whereas, diacetyl and acetoin produce a buttery aroma in the range between 1,300 and 1,400 ng/g. Phenylalanine causes the breakdown of methyl aldehydes and aldehydes producing a malty and fruity aroma while methyl acids produce a cheesy and sweaty aroma. Lipid degradation results in the formation of alkenes, alkanes, and aldehydes with straight chain, which have a green color and they have a rancid, metallic, and fruity flavor. In addition, 3- methyl butanal and ethanol (55 ppm) produce a fruity malty/mushroom flavor and a malty aroma, whereas methyl ketones are related with *Staphylococcus carnosus* and produce a cheesy, fruity and musty aroma. Methyl ketones and hexanal generated by linoleic acid oxidation also produces a green color and a fruity, metallic, musty, rancid, and cheesy aroma. On the other hand, 2-nonanone produced by ketones (2- pentanone) produces a green color and a mushroom-like fruity aroma. Ketones obtained from bacteria through chemical fermentation are enhanced by the growth of mold, especially ethyl esters metabolized from ethanol. Hence, the fruity flavors of fermented sausage are due to the presence of esters (83).

Ketones, hydrogen peroxides, aldehydes, and various different end products are produced from oxidation of lipids or by enzymatic or chemical reactions. Parameters affecting oxidation include unsaturated fatty acid content with reference to glycerol, content of oxygen, antioxidants (nitrite and spices) and peroxidants (salt and metals). Oxidation is also influenced by the various microbes affecting oxidative processes involved and rancid aroma (84).

The main differentiating parameter between non-fermented and fermented sausage is the presence of short-chain non-volatile fatty acids. Free fatty acids released from different lipases are associated with the complex flavor like non-volatile acid taste related with acetate and D-lactate. The compounds responsible for aroma production and pH are influenced by non-proteinaceous nitrogen. Researchers have found that ATP metabolite (hypoxanthine), proteolytic actions of raw materials with enzymes such as cathepsin D, proteolytic action of amines, and ammonia, sausages having a long drying time, branched aromatic aldehydes, acids (valine, isoleucine and leucine) and alcohols also affect taste (85).

Amino acids with branched chains present in sausages contain catabolites like aldehydes, branched-chain alcohol, and methyl acids. Researchers have conducted a study, in which triglycerides in association with endogenous and bacterial enzymes release free long-chain fatty acids of concentration ranging between 27 and 37 mg/g, where the ripening period length and type of raw material influence the quantity and rate of fatty acid release and contain less flavor (86).

## Fermentation Based on Bacterial Biofilms

The EPS matrix of biofilms mostly contains extracellular polysaccharides, extracellular proteins, and extracellular DNA (eDNA). All the complex constituents are responsible for determining biofilm structure and allowing bacteria residing in biofilms to adapt to altered environmental conditions (87). Recently, scientists have discovered that cells within the biofilms are capable of growing steadily with increased activity thereby providing conditions favorable for industrial fermentation (88). Therefore, cells residing within biofilms could withstand harsh environments like rise in osmotic pressure, lack of oxygen, and increased density of cells during fermentation. Hence, fermentation with immobilized biofilm was designed and applied for industrial fermentation to improve the absorption capability of strains (89).

During biofilm-mediated fermentation, the cells can tolerate agitation at a high speed. In addition to this, biofilm cells, which remain attached to the surface of the carriers can be changed while replacing the fermentation broth with fresh medium (90). Because of the higher activity of cells, seed culture may be removed and the lag phase can be decreased substantially shortening the production cycle (91).

Within biofilms, extracellular DNA (eDNA) plays a major role in the linking between cells, carriers, proteins, and extracellular polysaccharides. eDNA influences the structure of biofilm in *Staphylococcus aureus* and *Streptococcus mutans* (92). Researchers have observed that eDNA can be woven with the help of extracellular polysaccharides for the formation of a flocculent inseparable structure in the biofilms of *S. mutans* (93). Apart from this, eDNA can also maintain the extracellular matrix of biofilm and can serve as a “kite-line” restricting the external flow of bacteria at the top of the structure of biofilm. eDNA plays a significant role in the complete biofilm life cycle from the initial fermentation phase (formation) to dissociation (94). Thus, eDNA can be considered as the main component in the formation of biofilm as well as the main ingredient responsible in maintaining the structure of biofilm. So, increased accumulation of eDNA is advantageous for formation of biofilm. Extracellular nuclease is one of the enzymes that can bring about eDNA degradation. It was found that the elimination of genes encoding for nuclease *exeS* and *exeM* can facilitate eDNA accumulation and formation of biofilm in *Shewanella oneidensis*, strain MR-1 (95). On contrary, several bacteria possess type IV secretion systems (T4SS), which can secrete DNA-protein or DNA (96). Inside T4SS, protein family *VirB11* was the most significant for protein and DNA to go through.

Biofilm-immobilized fermentation is not much prevalent one of the important strains of *Corynebacterium glutamicum* for the production of amino acids. The reason behind this is poor ability of adsorption on materials or formation of EPS in *C. glutamicum*. Researchers have engineered a strain of *C. glutamicum* and named it as Pro-Δ*exeM*, in which the extracellular gene *exeM* encoding for nuclease was eliminated for effective increase in the quantity of eDNA within EPS and cellular adhesion on the materials used as carriers (97). In repeated biofilm-immobilized batch fermentation, production of L-proline increased to 17.1 g/L from 10.2 g/L. In short, from this research, it was indicated that a synthetic biofilm of *C. glutamicum* can be favorably used in the production of L-proline and can also be used in other industries and this same biological engineering may be applied in engineering other microbial strains.

Previously, the original strain of *C. glutamicum* ATCC 13032- ProB was used in the establishment of biofilm-mediated fermentation in L-proline production. However, this strain was used to form less amounts of biofilms and so it could not be effectively used in fermentation industries.

The biofilm-based fermentation technology is applied widely in industries (98). The strain of Pro-Δ*exeM*1 has a better capability to form biofilm and so it was chosen for the production of L-proline because it led to a greater production of L-proline through immobilized fermentation in comparison to other bacterial strains (97). Biofilm carrier addition is the most significant in biofilm-immobilized fermentation. This is because bacteria will get fixed to the carrier surfaces to support the development of biofilms. However, carrier selection must be dependent on whether it will be suitable for mass and oxygen transfer during the cellular growth process. The bacterial cells present within the fermentation broth were largely decreased facilitating the ease of separation of the final product from the fermentation broth. In comparison with planktonic cell fermentation, the carrier with immobilized biofilm acted as an immobilized biocatalyst, which can be repeatedly reused. Thus, seed culture was not needed.

### Use of EDNA for Enhanced Formation of Biofilm

**Figure 2:**
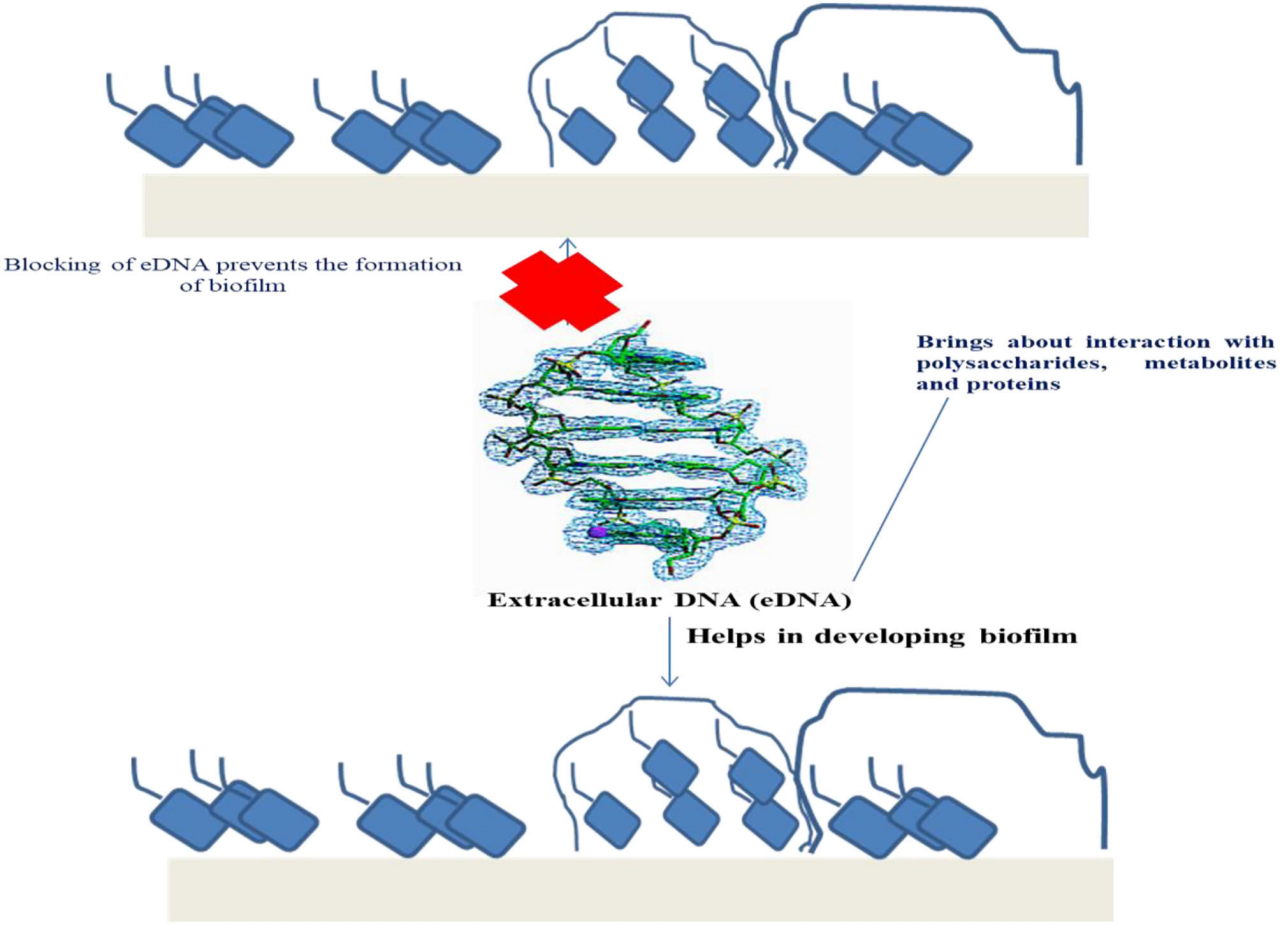
Role of eDNA in the development and regulation of biofilm.

Wild strains of bacteria form less biofilms because of the eDNA hydrolysis by the action of extracellular DNase I during the process of biofilm formation or the biofilm could not develop because of inadequate eDNA for the initial phases of biofilm formation. Within the culture medium, various concentrations and lengths of the DNA were added externally to facilitate biofilm formation (99). This demonstrated that exogenous DNA addition can have a positive impact on biofilm formation process. Particularly, a very low concentration of 0.6 ng/ μl of 5,000 bp long DNA fragments can help in the formation of large amounts of biofilm (97) (Figure 2).

### Molecular Genetic Modifications

The homologs of the nuclease genes of bacterial strain were identified through NCBI protein BLAST and then the *VirB11* gene of the test organism (wild type strain) was searched for in NCBI. *exeM* knockout and *VirB11* overexpression led to remarkable enhancement in biofilm formation. It was unknown that the Pro-Δ*exeM*1 knockout strain eliminated 1 or 2 *exeM* genes. All *exeM* genes from Pro-Δ*exeM*1 strain were removed.

Moreover, the Pro-Δ*exeM*1 strain helped in producing the highest amount of biofilms and eDNA indicating that the reduction of extracellular nucleases may prove more effective than promoting the eDNA secretion *via VirB11* (100).

### Applications of Biofilm in Fermented Food

The development of biofilm can be a beneficial process as it may lead to the improvement of the biochemical quality, texture properties, flavors, and taste of the food products (101). The polymicrobial interactions existing within the biofilm help in inducing certain food processes. The mechanism of cheese ripening and production of beverages can be efficiently carried out in the presence of microbial interactions (102). Various types of microflora are associated with different types of fermented food products that include various meat, vegetable, and dairy products. It has been observed that throughout the world the biofilmed form of fermented food products comprise of mixed biofilm are those associated to the surfaces, e.g., cheese, biofilm that remains suspended within liquids like vinegar, biofilm exhibiting dispersed growth within liquids like yogurt and natural wine or those associated with various types of semi-solid like miso and kimchi (10). The production is wine is associated with the mixture of biofilm comprising of bacteria, fungi, and yeast that helps in the ripening of grapes. The cellular communication existing within the biofilm plays a vital role in the process of wine production (103–105). *Lactobacillus plantarum* plays an important role as model organism for the development of wine (28). The use of organisms like *Lactobacillus delbrueckii, Streptococcus thermophiles, Lactobacillus lactis*, and *Enterococcus faecium* play a vital role in the process of production of cheese (106, 107). The use of biofilm formed by non-started lactic acid bacteria that remain adhered to wooden plank or wooden vat plays a vital role in the process of traditional stretched cheese. The process of cheese ripening is also associated with beneficial biofilm forming bacteria (108). The seasonal profiling of the cheese can be also associated with the use of certain microbial biofilms, making it attractive for the consumers (109). The biofilm formed by yeast and lactic acid bacteria can be used for the purpose of producing olive (110). The use of the biofilm formed by *P. membranifaciens* and *L. pentosus* can be used in the process of submerged fermentation for the production of black olives (111, 112).

### Transcriptomic Analysis for Improved Formation of Biofilms

*Escherichia coli* was biologically engineered for *fimH* gene over-expression thereby enhancing its ability to form biofilms under aerobic and industrial conditions of cultivation (113). L-threonine is an essential amino acid required by the human body and it is widely in demand in food, pharmaceutical, and chemical industries (114). Nowadays, fermentation with microbes is increasingly used for industrial production of L-threonine by taking *E. coli* as the best strain (115). Fermentation of L-threonine was carried out so far in planktonic-cell batch mode of fermentation, where cells were not reused following fermentation. Batch fermentation and absence of cell reusability increase the production cost and decrease productivity. The free cells remain dispersed within the fermentation medium and are frequently challenged with stresses like shear forces when aerobic fermentation takes place leading to reduced viability of cells used in the process of fermentation. These limitations need to be addressed and solved immediately for improving the efficiency of fermentation. As an option, biofilm-mediated immobilized fermentation has been used as an alternate method to free-cell fermentation due to its advantages like protection with matrix of biofilm, increased metabolic actions and cell reusability in comparison to free-cell fermentation (116). Biofilms of some microbes like *C. glutamicum, Clostridium acetobutylicum, S. cerevisiae*, and *Aspergillus niger* can be used for continuous (repeated batch fermentation) or batch fermentation efficiently (117).

Type I fimbriae is one of the most significant factors helping in the formation of biofilm of Gram negative bacteria like *E. coli* (6). In *E. coli*, a protein encoded by *fimH* gets secreted and is present at the type I fimbriae top playing a key role in biofilm structure production and acts as an adhesion (118). Cells utilize such structure for nutrient absorption and for overcoming shear forces. It was also found that biofilm cells of *E. coli* can tolerate more harsh conditions (119).

*E. coli* was at first engineered metabolically for *fimH* gene overexpression for enhancing the formation of biofilm (113). They created a biofilm-immobilized fermentation by the use of a carrier for providing support to the biofilm. Cells on adhesion with the carrier surface produced a huge quantity of biofilm, which can tolerate shaking at a high-speed. However, the cells of the biofilm, which get attached to the surface of the carrier, may be reused after replacing the fermentation broth with freshly prepared media (91). Because of increased cellular activities and the reuse of cells in biofilm-mediated fermentation, there is no requirement of seed culture and so the period of fermentation and lag phase of the cells get decreased consequentially. Moreover, this experiment presented a successful instance of preparing a biofilm-based fermentation in presence of aerobic conditions for the effective production of biochemicals.

In order to study the underlying mechanism behind the increased formation of biofilm, transcriptome analysis of the engineered and the wild type gene of the bacteria need to be performed. The gene expression ratio of the genes that participated in the enhanced biofilm production display remarkable differences among various strains and the genes, which need to be regulated, may be classified into 6 different groups. In the study with biofilm production from *E. coli*, it was found that the gene *fimH* (encodes type I adhesin of fimbriae) and the gene cluster *flu* help in adhesin secretion thereby adhering the cells to the surfaces (120). *FlhC* and *FlhD* are activators of transcription, which take part in assembly and regulation of the flagella giving the cell motility and enhancing the formation of biofilms (121). *CsgD* in combination with *csgC, csgB*, and *csgA* is responsible for the assembly of curli genes, biosynthesis of structural components and their transport for the formation of biofilm (120). The *glgP, glgC*, and *glgA* regulate the biosynthesis of glycogen (121). As the cellular density reaches the threshold level, *lsrR, speD, metK*, and *luxS* assist in the biosynthesis of signal molecule autoinducer 2 (AI-2) involved in quorum sensing (QS) thereby activating the transcriptional factors required for biofilm formation initiation (122). The gene *luxS* is actually involved in catalyzing the reaction of AI-2 and it is widely found in both Gram positive as well as Gram negative bacteria thereby indicating an increased homologous conservation. All of these genes get up-regulated up to varying extents in the mutated strains of bacteria while they remain down-regulated in the wild type bacterial strain. Extracellular AI-2 concentration can be rapidly reduced by the ATP binding cassette (ABC) transporter of the gene *lsr*, which transports AI-2 within the cell. After transcriptome data analysis, it can be concluded that some of the genes of the *lsr* operon get down-regulated thereby resulting in increased extracellular AI-2 accumulation leading to the expression of genes involved in biofilm formation (123). These genes display varying up-regulation degrees in the genetic overexpression of the biofilm-related genes in the engineered strain and this may be validated using qRT-PCR.

### Application of Next Generation Sequencing (NGS)/Omics in Analysis of Engineered Biofilms Used in Food Fermentation

**Figure 3:**
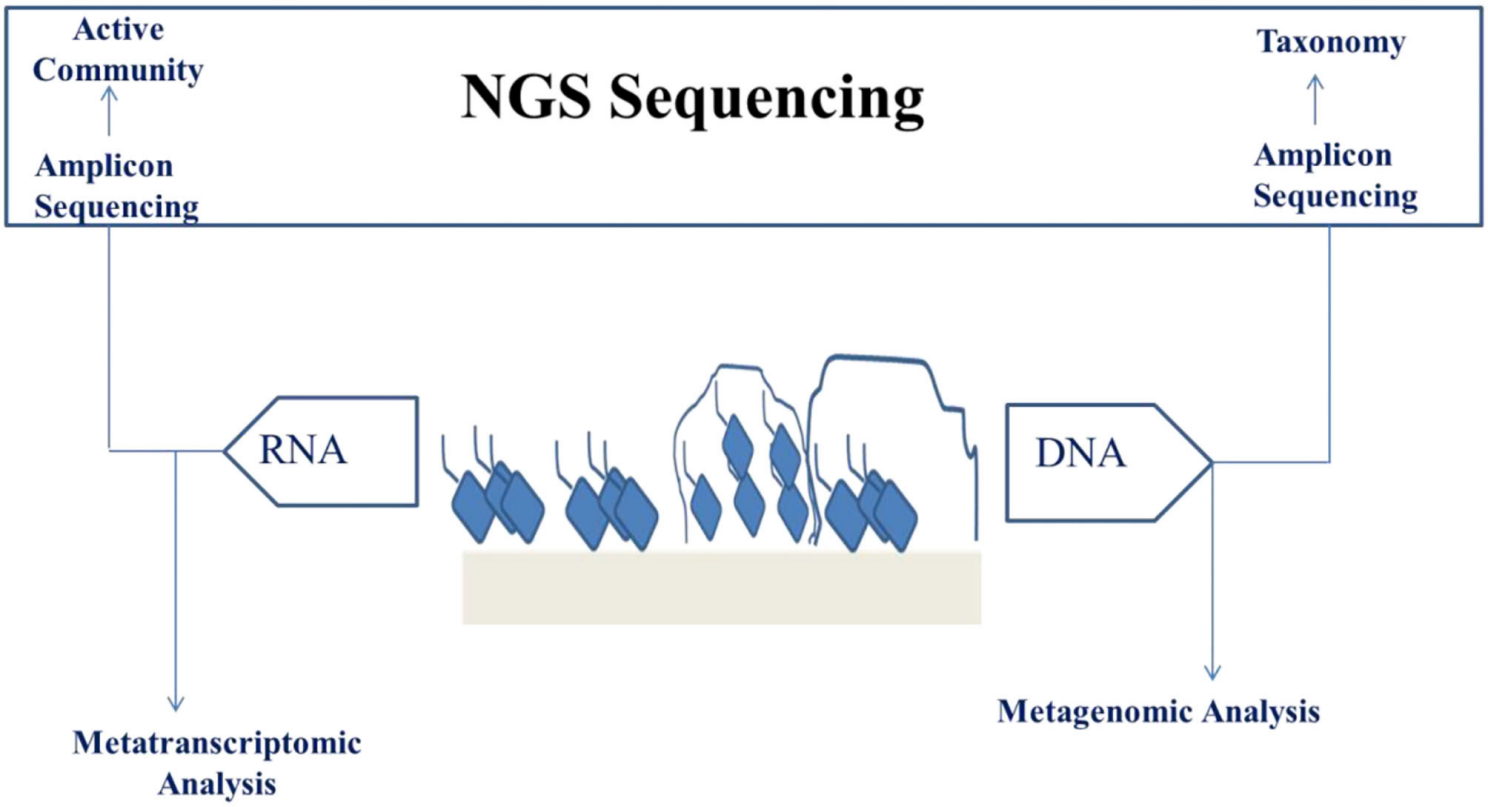
NGS analysis of biofilm for the purpose of validating engineered food.

Genome sequencing of microbes has become a significant branch in the stream of food microbiology because of the increased improvements as well as affordability of the sequencing speed and data quality. This is a result of the advancements in the sequencing techniques, which is together called NGS. NGS comprises the immensely single and parallel sequencing of molecules providing both long and short sequence reads, respectively. Short-read sequences are mostly accurate and gives read lengths within 100–300 bp, which are combined to form incomplete/draft genomes. Complete genomes cannot be produced from short read sequences because of complexity in arranging repetitive portions and large rearrangements of the genomes like inversions, deletions, and insertions. For numerous uses, including phylogeny and comparative genomics, this will not present any problem but when complete genomes are needed and for complex genetic region determination, long sequence reads are required. Although long reads produce reads of 10–50 kb length, there exist the highest rate of errors (124). Nowadays, sequencing of microbial DNA can be carried out on various platforms like Ion Torrent, Illumina, Nanopore, and PacBio (Figure 3).

## Conclusion

All over the world, for thousands of years, fermented food has become a part of the human diet because of the alterations brought naturally in them for enhancing nutritional qualities as well as flavor by just knowing the functions performed by various microbes.

A biofilm-based immobilized system of fermentation for amino acid production in large scale in industries possess several advantages. The biologically engineered strains can successfully increase the biofilm formation rate under cultivating conditions of industries and this process can be even applied in repeated-batch (continuous) immobilized fermentation. Analysis of transcriptome profiles demonstrated that formation of biofilms can be increased by regulating the genes involved in biofilm formation and development.

Application of NGS in food quality improvement has been thought to be a game changer for food industries worldwide. There is an urgent need in bridging the technological gap existing between theoretical application and field-application of these NGS strategies. Moreover, extension of NGS impacts will result in a reduced cost in food industries. This is because the cost to produce biologically engineered genomic sequences of bacteria will diminish rapidly and within a few years, the expense of the application of NGS technology will out-smart the costs of microbial cultures and their physiological analyses. This expense reduction is an additional benefit that this new technology has been designed to deliver.

## Author Contributions

SG, MN, DL, and TS conceived and designed the experiments. SP, ZK, NN, HE, and RR were responsible for writing—original draft preparation. SG, MN, DL, TS, SP, ZK, NN, HE, and RR contributed to the formatting and editing according to journal guidelines and writing—review and editing. All authors have read and agreed to the published version of the manuscript. All authors contributed to the article and approved the submitted version.

## Funding

This work was supported by National Conservation Trust Fund for Natural Resources from Ministry of Energy and Natural Resources Malaysia (grant no: 304/PPSK/6150219).

## Conflict of Interest

SP was employed by NatNov Bioscience Private Limited. The remaining authors declare that the research was conducted in the absence of any commercial or financial relationships that could be construed as a potential conflict of interest.

## Publisher's Note

All claims expressed in this article are solely those of the authors and do not necessarily represent those of their affiliated organizations, or those of the publisher, the editors and the reviewers. Any product that may be evaluated in this article, or claim that may be made by its manufacturer, is not guaranteed or endorsed by the publisher.
